# Magnesium deficiency and long-term incident dementia among COVID-19 survivors: a propensity score-matched cohort study

**DOI:** 10.3389/fnut.2026.1871623

**Published:** 2026-07-01

**Authors:** I-Yin Hung, Wei-Ting Wang, Ping-Hsin Liu, Wan-Jung Cheng

**Affiliations:** 1 Department of Anesthesiology, Chi Mei Medical Center, Tainan City, Taiwan; 2 Department of Anesthesiology, E-Da Hospital, I-Shou University, Kaohsiung City, Taiwan; 3 Department of Anesthesiology, Chi Mei Medical Center, Liouying, Tainan City, Taiwan

**Keywords:** COVID-19, dementia, magnesium deficiency, mild cognitive impairment, neurodegeneration, propensity score matching

## Abstract

**Background:**

COVID-19 survivors face an elevated risk of long-term cognitive decline, yet potentially modifiable factors that shape post-COVID dementia trajectories remain insufficiently characterized. Low magnesium status has been linked to dementia risk in general populations, but whether low magnesium status is associated with long-term dementia risk among COVID-19 survivors remains unclear.

**Methods:**

This propensity score–matched retrospective cohort study used the TriNetX Global Collaborative Network to examine the association between low serum magnesium ( < 1.7 mg/dL on two measurements) and incident dementia among hospitalized COVID-19 survivors aged ≥ 55 years (2020–2023). A 1-year landmark period was imposed to reinforce temporal separation between exposure and outcome. The primary outcome was overall incident dementia over 5 years. Secondary outcomes included Alzheimer’s disease, vascular dementia, other dementia types, and mild cognitive impairment.

**Results:**

After matching, 58,991 patients with low magnesium and 58,991 controls were included. Magnesium deficiency was associated with a higher risk of overall dementia [2.12% vs. 1.77%; hazard ratio (HR) 1.39, 95% confidence interval (CI) 1.28–1.51; *P* < 0.001; *E*-value 2.13). Among secondary outcomes, low magnesium was associated with higher risks of Alzheimer’s disease (HR 1.34; *P* = 0.005), vascular dementia (HR 1.50; *P* = 0.001), other dementia types (HR 1.38; *P* < 0.001), and mild cognitive impairment (HR 1.27; *P* < 0.001). The association between magnesium deficiency and overall incident dementia persisted across sensitivity analyses and sex-stratified subgroups.

**Conclusion:**

In this observational study, low serum magnesium was associated with increased long-term dementia risk among COVID-19 survivors. Because eligibility required repeated magnesium testing and follow-up healthcare visits were marginally more frequent in the low-magnesium group, selection bias and differential dementia detection cannot be excluded. These hypothesis-generating findings warrant prospective validation.

## 1 Introduction

Although the acute phase of the COVID-19 pandemic has largely subsided, its long-term neurocognitive consequences are increasingly being recognized (1–3). Large-scale cohort studies and meta-analyses have reported higher risks of cognitive impairment and incident dementia after COVID-19, with risk trajectories described for up to 2 years after infection (4–7). Given the large number of individuals infected with SARS-CoV-2 worldwide (8–10), even a modest excess risk of dementia could translate into a substantial public health burden, particularly among hospitalized survivors who may have experienced acute neuroinflammation, microvascular injury, and metabolic dysregulation (11–13). However, existing research has primarily addressed whether COVID-19 itself is associated with subsequent dementia (4–7), whereas potentially modifiable factors that may shape long-term dementia trajectories after COVID-19 remain insufficiently characterized. Given that dementia is a progressive and largely irreversible condition with substantial individual, caregiver, and health-system burdens, identifying modifiable risk factors for dementia among COVID-19 survivors remains an important public health priority.

Magnesium may be biologically relevant to post-infectious neurodegeneration because it contributes to synaptic plasticity, regulates N-methyl-D-aspartate receptor activity, modulates excitotoxicity, and may attenuate neuroinflammatory signaling (14–17). Consistent with this mechanistic rationale, studies in general-population cohorts and stroke populations have identified low magnesium status as a potential risk factor for dementia or cognitive impairment (18–23), supporting the hypothesis that magnesium deficiency may mark or contribute to long-term cognitive vulnerability. However, because most existing studies were conducted in the general population(18–23), whether low magnesium status is associated with long-term dementia risk among COVID-19 survivors remains uncertain. This question is clinically relevant because COVID-19 survivors may experience converging mechanisms of neural injury, including possible viral neuroinvasion, systemic inflammatory activation, endothelial dysfunction, blood–brain barrier disruption, and cerebral microvascular injury (24–28). To date, evidence directly linking magnesium status to post-COVID cognitive outcomes is limited. Guzmán-Esquivel et al. (29). studied 111 older adults over 6 months and reported hypomagnesemia as a predictor of cognitive impairment; however, large-scale studies with longer follow-ups are needed.

Therefore, using a large multicenter electronic health record database, we examined the association between low serum magnesium levels and long-term incident dementia among hospitalized COVID-19 survivors, applying a 1-year landmark design to reinforce temporal separation between exposure and outcome ascertainment.

## 2 Materials and methods

### 2.1 Data source and ethical statement

This propensity score–matched retrospective cohort study used de-identified electronic health records available through the TriNetX Global Collaborative Network, a federated research platform that links data from 173 healthcare organizations. The TriNetX database has been widely used in epidemiological and real-world evidence studies across multiple clinical fields (30–32). Because only aggregated, de-identified information is accessible through TriNetX, the need for individual informed consent was waived. Ethical approval was granted by the Institutional Review Board of Chi Mei Medical Center, and all procedures adhered to the Declaration of Helsinki and the applicable institutional regulations.

### 2.2 Exposure definition

Adults aged ≥ 55 years with documented COVID-19 between January 1, 2020, and December 31, 2023, were eligible for inclusion. COVID-19 was identified using either an ICD-10-CM diagnosis code for COVID-19 (U07.1) or a SARS-CoV-2–related RNA laboratory record. To restrict the analysis to clinically meaningful infections, only patients who were hospitalized during the COVID-19 episode were included in the study.

Patients were classified according to repeated serum magnesium measurements rather than a single laboratory measurement. Serum magnesium was identified using the magnesium mass concentration in serum or plasma (LOINC: 19123-9). The magnesium deficiency cohort included patients with two qualifying serum magnesium measurements < 1.7 mg/dL within a 1-year interval. The reference cohort included patients with two qualifying serum magnesium measurements within the normal range (1.70–2.20 mg/dL) within a 1-year interval. For both cohorts, the index date was defined as the date of the second qualifying measurement of magnesium. This design ensured that magnesium status was established before the start of follow-up and reduced exposure misclassification related to transient magnesium abnormality. To prevent acute COVID-19 illness from confounding magnesium levels, only patients whose COVID-19 episode preceded the index date by at least 6 months were included.

### 2.3 Exclusion criteria

Patients were excluded if they had evidence of dementia or related neurodegenerative disorders before the index date, including vascular dementia (ICD-10-CM: F01), dementia in other diseases classified elsewhere (F02), unspecified dementia (F03), Alzheimer’s disease, and other degenerative diseases of the nervous system (G30–G32). To reduce confounding from conditions strongly associated with magnesium dysregulation, baseline cognitive impairment, or competing neurologic risk, patients were also excluded if they had end-stage renal disease (N18.6), stage 4 or stage 5 chronic kidney disease (N18.4–N18.5), dependence on renal dialysis (Z99.2), bipolar disorder (F31), schizophrenia spectrum or other psychotic disorders (F20–F29), Parkinson’s disease (G20), bariatric surgery status (Z98.84) or bariatric surgery procedures, cerebral infarction (I63), or nontraumatic intracerebral hemorrhage (I61) before the index date.

A 1-year landmark period was imposed to reinforce temporal ordering between magnesium exposure and incident dementia. Patients who were documented as deceased before the start of follow-up were also removed to ensure that all eligible individuals actually entered the observation window.

### 2.4 Data collection and propensity score matching

To improve comparability between patients with persistent magnesium deficiency and those with persistently normal magnesium levels, we performed 1:1 propensity score matching using a greedy nearest-neighbor approach with a caliper width of 0.1 pooled standard deviations. The propensity score was estimated from prespecified covariates selected for their potential associations with both serum magnesium status and dementia risk. These covariates included demographic factors, cardiometabolic and vascular diseases, neuropsychiatric comorbidities, medication use, and laboratory indices reflecting kidney function, inflammation, nutrition, glucose metabolism, hematologic status, and body composition. All matching variables are listed in Supplementary Table 1. Covariate balance after matching was evaluated using standardized mean differences (SMDs); an absolute SMD < 0.10 indicated an acceptable balance. The distribution of propensity scores before and after matching was visualized using density plots.

### 2.5 Primary and secondary outcomes

The main outcome was incident dementia, defined as a new diagnosis of vascular dementia, dementia in other diseases, unspecified dementia, or Alzheimer’s disease (ICD-10-CM codes: F01, F02, F03, or G30). Outcome ascertainment began after the completion of the 1-year landmark period and continued for up to 5 years thereafter. Secondary outcomes included specific dementia phenotypes, including Alzheimer’s disease (G30), vascular dementia (F01), other or unspecified dementia (F02 or F03), and mild cognitive impairment (G31.84). Follow-up ended at the first occurrence of the relevant outcome, death, last recorded clinical encounter, or completion of the 5-year observation period.

### 2.6 Validation of study design

Prespecified control analyses were conducted to evaluate whether the observed associations were consistent with expected biological and epidemiological patterns. Hypocalcemia, defined as a serum calcium level of < 8.5 mg/dL, was selected as a positive control outcome because magnesium depletion can disrupt calcium regulation and parathyroid hormone activity. Acute appendicitis (ICD-10-CM: K35) was used as a negative control outcome, as it was not expected to be related to the baseline magnesium status through a plausible biological pathway. To assess the possibility of differential outcome detection, we also compared the follow-up healthcare utilization between the groups using hospital visit frequency. In addition, recurrent or subsequent COVID-19 diagnoses during follow-up were examined to determine whether between-group differences in reinfection burden contributed to differences in the long-term risk of dementia.

### 2.7 Sensitivity analyses, subgroup analyses, and multivariable regression

Robustness was evaluated using three prespecified sensitivity analyses. First, the cohort was restricted to patients who required intensive care after COVID-19, representing a subgroup with more severe acute infections. Second, patients who died during follow-up were excluded to determine whether the association persisted among long-term survivors. Third, analyses were repeated in patients older than 65 years to assess whether the findings were consistent in an age group with a higher baseline risk of dementia.

Sex-specific subgroup analyses were conducted separately for men and women. Within each stratum, propensity score matching and outcome analyses were repeated independently to preserve the covariate balance within the subgroup comparisons. Effect modification by sex was examined using interaction tests. In addition, sequential multivariable Cox proportional hazards models were fitted to evaluate whether magnesium deficiency was associated with incident dementia after stepwise adjustment for potential confounders.

### 2.8 Statistical analysis

Time-to-event analyses were performed using cause-specific Cox proportional hazard models, with effect estimates reported as hazard ratios (HRs) and 95% confidence intervals (CIs). We used a cause-specific framework because the primary objective was to examine the etiological association between magnesium status and subsequent dementia, rather than to predict cumulative incidence while accounting for death as a competing event. Accordingly, death was treated as a censoring event in the primary cause-specific analysis, and the resulting HRs should be interpreted as associations with the instantaneous rate of dementia among patients who remained alive and under observation, rather than as direct estimates of absolute cumulative incidence. The proportional hazards assumption was evaluated using Schoenfeld residuals. Dementia-free survival was visualized using Kaplan–Meier curves, and between-group differences were assessed using log-rank tests.

For the primary endpoint, E-values were calculated to estimate the minimum magnitude of association that an unmeasured confounder would need to have with both magnesium deficiency and dementia to negate the observed association between them. Statistical significance for the primary analysis was defined as a two-sided α level of 0.05. Analyses of secondary endpoints, control outcomes, sensitivity models, and subgroups were considered exploratory, and no adjustments for multiple comparisons were applied. All analyses were conducted using the available data without imputation.

## 3 Results

### 3.1 Patient selection and baseline characteristics

**FIGURE 1:**
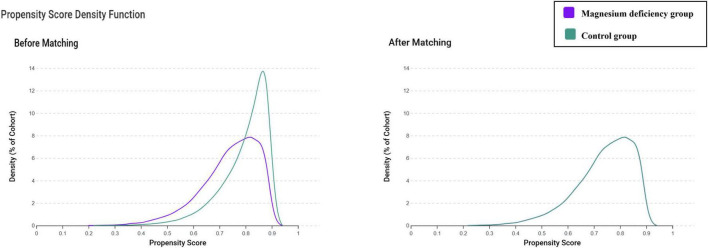
Propensity score density distributions before and after matching. The figure shows propensity score density distributions for hospitalized COVID-19 survivors with low magnesium status and controls with normal magnesium status before and after 1:1 propensity score matching. Before matching, the distributions showed imbalance between groups. After matching, the distributions demonstrated substantial overlap, indicating improved covariate balance between the low-magnesium and control cohorts.

A total of 59,010 COVID-19 survivors met the criteria for low serum magnesium levels, and 228,617 patients constituted the initial reference cohort with normal magnesium levels. After 1:1 propensity score matching, 58,991 patients remained in each group. The mean age was approximately 67 years, roughly 53% were female, and approximately 70% self-identified as white. Before matching, notable imbalances were observed in several covariates, such as hypertension, neoplasms, diabetes mellitus, anxiety disorder, and chronic kidney disease. Following matching, all standardized mean differences fell below 0.10, indicating an adequate covariate balance across demographic, comorbidity, laboratory, and medication variables (Table 1). The propensity score density distributions confirmed a satisfactory overlap between the two cohorts (Figure 1).

**TABLE 1:** Baseline characteristics of hospitalized COVID-19 survivors with low magnesium status and matched controls before and after propensity score matching.

Variables	Before matching			After matching		
	Low Mg group (*n* = 59,010)	Control group (*n* = 228,617)	SMD	Low Mg group (*n* = 58,991)	Control group (*n* = 58,991)	SMD
Patient characteristics						
Age at index (years)	66.7 ± 9.7	67.6 ± 10.2	0.083	66.7 ± 9.7	66.7 ± 9.9	0.002
BMI ≥ 30 (kg/m^2^)	27,514 (46.6)	101,182 (44.3)	0.048	27,508 (46.6)	27,593 (46.8)	0.003
Female	31,508 (53.4)	115,740 (50.6)	0.055	31,499 (53.4)	31,788 (53.9)	0.010
White	41,298 (70.0)	163,780 (71.6)	0.036	41,289 (70.0)	41,300 (70.0)	0.000
Black or African American	10,495 (17.8)	36,426 (15.9)	0.049	10488 (17.8)	10510 (17.8)	0.001
Asian	1,168 (2.0)	6,140 (2.7)	0.047	1,168 (2.0)	1,130 (1.9)	0.005
Comorbidities and healthcare utilization						
Factors influencing health status and contact with health services	49,570 (84.0)	179,557 (78.5)	0.140	49,551 (84.0)	49,588 (84.1)	0.002
Essential (primary) hypertension	43,458 (73.6)	151,736 (66.4)	0.159	43,442 (73.6)	43,514 (73.8)	0.003
Neoplasms	28,237 (47.9)	95,888 (41.9)	0.119	28,225 (47.9)	28,352 (48.1)	0.004
Diabetes mellitus	26,736 (45.3)	71,915 (31.5)	0.288	26,724 (45.3)	27,184 (46.1)	0.016
Ischemic heart diseases	21,725 (36.8)	78,030 (34.1)	0.056	21,719 (36.8)	21,617 (36.6)	0.004
Overweight and obesity	19,325 (32.7)	66,719 (29.2)	0.077	19,321 (32.7)	19,440 (33.0)	0.004
Anxiety, dissociative, stress-related, somatoform and other nonpsychotic mental disorders	18,772 (31.8)	61,090 (26.7)	0.112	18,764 (31.8)	18,718 (31.7)	0.002
Sleep disorders	17,584 (29.8)	61,733 (27.0)	0.062	17,580 (29.8)	17,553 (29.8)	0.001
Mood [affective] disorders	16,380 (27.8)	51,114 (22.4)	0.125	16,371 (27.8)	16,391 (27.8)	0.001
Nicotine dependence	15,202 (25.8)	46,037 (20.1)	0.134	15,189 (25.8)	15,129 (25.6)	0.002
Heart failure	14,468 (24.5)	47,905 (21.0)	0.085	14,462 (24.5)	14,621 (24.8)	0.006
Diseases of liver	14,107 (23.9)	34,783 (15.2)	0.220	14,092 (23.9)	13,750 (23.3)	0.014
COPD	13,863 (23.5)	44,603 (19.5)	0.097	13,853 (23.5)	13,903 (23.6)	0.002
Disorders of thyroid gland	13,622 (23.1)	48,214 (21.1)	0.048	13,617 (23.1)	13,553 (23.0)	0.003
Chronic kidney disease (CKD)	12,351 (20.9)	35,715 (15.6)	0.138	12,340 (20.9)	12,241 (20.8)	0.004
Atrial fibrillation and flutter	12,134 (20.6)	45,119 (19.7)	0.021	12,132 (20.6)	12,069 (20.5)	0.003
Vitamin D deficiency	10,099 (17.1)	35,639 (15.6)	0.041	10,094 (17.1)	10,093 (17.1)	0.000
Alcohol related disorders	8,529 (14.5)	17,155 (7.5)	0.224	8,510 (14.5)	8,263 (14.0)	0.012
COVID-19	7,623 (12.9)	23,820 (10.4)	0.078	7,617 (12.9)	7,336 (12.4)	0.014
Cerebrovascular diseases	7,370 (12.5)	25,312 (11.1)	0.044	7,369 (12.5)	7,302 (12.4)	0.003
Malnutrition	70,05 (11.9)	12,366 (5.4)	0.232	6,988 (11.9)	6,561 (11.1)	0.023
Systemic connective tissue disorders	2,208 (3.7)	7,814 (3.4)	0.017	2,208 (3.7)	2,228 (3.8)	0.002
Intracranial injury	1,571 (2.7)	5,102 (2.2)	0.028	1,570 (2.7)	1,569 (2.7)	0.000
Laboratory data						
Hemoglobin ≥ 12 g/dL	48,263 (81.8)	184,093 (80.5)	0.032	48,248 (81.8)	48,368 (82.0)	0.005
Albumin ≥ 3.5 g/dL	48,463 (82.1)	174,446 (76.3)	0.144	48,445 (82.1)	48,680 (82.5)	0.010
HbA1c ≥ 9%	7,423 (12.6)	17,214 (7.5)	0.169	7,419 (12.6)	7,499 (12.7)	0.004
eGFR ≥ 60 mL/min/1.73 m^2^	48,752 (82.6)	177,280 (77.5)	0.127	48,734 (82.6)	48,952 (83.0)	0.010
C-reactive protein ≥ 10 mg/L	14,020 (23.8)	36,358 (15.9)	0.198	14,009 (23.8)	13,896 (23.6)	0.005
Vitamin B12 300–900 pg/mL	13,375 (22.7)	39,190 (17.1)	0.139	13,365 (22.7)	13,354 (22.6)	0.000
Medications						
Central nervous system medications	54,346 (92.1)	197,771 (86.5)	0.182	54,327 (92.1)	54,333 (92.1)	0.000
Benzodiazepine	39,612 (67.1)	128,878 (56.4)	0.223	39,593 (67.1)	39,974 (67.8)	0.014
Proton pump inhibitors	34,499 (58.5)	103,611 (45.3)	0.265	34,481 (58.5)	34,616 (58.7)	0.005
Diuretics	31,474 (53.3)	98,383 (43.0)	0.207	31,457 (53.3)	31,405 (53.2)	0.002
Magnesium	29,465 (49.9)	82,588 (36.1)	0.282	29,446 (49.9)	30,168 (51.1)	0.024
Insulins and analogues	24,644 (41.8)	60,788 (26.6)	0.324	24,629 (41.8)	24,788 (42.0)	0.005
Anticonvulsants	21,522 (36.5)	65,943 (28.8)	0.163	21,513 (36.5)	21,543 (36.5)	0.001
Blood glucose lowering drugs, excl. Insulins	17,366 (29.4)	43,518 (19.0)	0.244	17,359 (29.4)	17,574 (29.8)	0.008
Anticholinergics	12,835 (21.8)	39,955 (17.5)	0.108	12,829 (21.8)	12,989 (22.0)	0.007

Data are presented as n (%) or mean ± standard deviation. BMI, body mass index; CKD, chronic kidney disease; COPD, chronic obstructive pulmonary disease; COVID-19, coronavirus disease 2019; eGFR, estimated glomerular filtration rate; HbA1c, hemoglobin A1c; Mg, magnesium; SMD, standardized mean difference.

### 3.2 Primary and secondary outcomes

**FIGURE 2:**
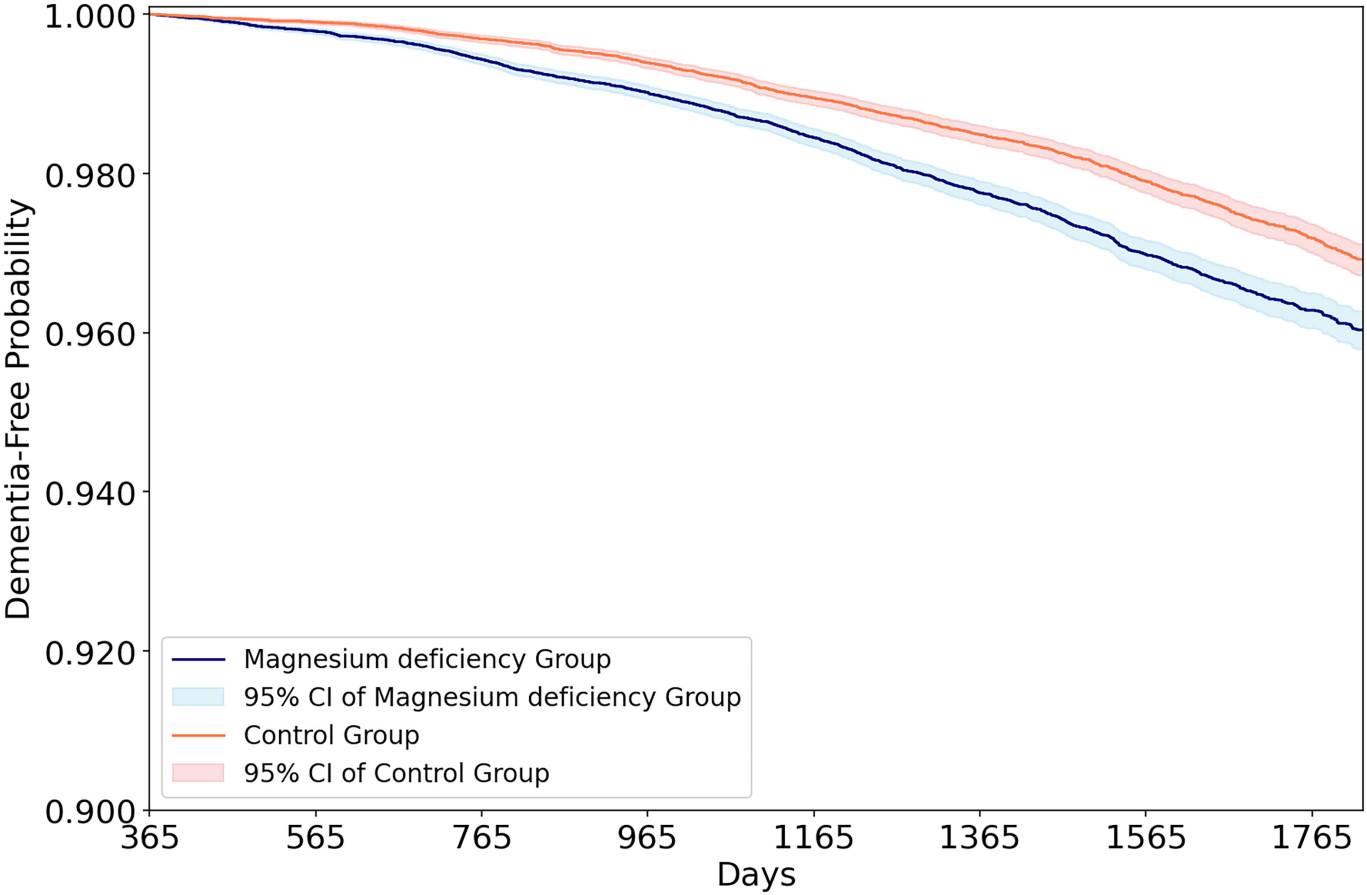
Kaplan–Meier curves for dementia-free survival after propensity score matching. Kaplan–Meier curves compare dementia-free survival between hospitalized COVID-19 survivors with low magnesium status and matched controls with normal magnesium status. Outcome ascertainment began after completion of the 1-year landmark period and continued for up to 5 years. Shaded areas represent 95% confidence intervals. Patients with low magnesium status had significantly lower dementia-free survival than matched controls during follow-up (HR 1.39, 95% CI 1.28–1.51, *p* < 0.001). COVID-19, coronavirus disease 2019; HR, hazard ratio; Mg, magnesium.

Over the 5-year follow-up period after the 1-year landmark, the mean follow-up duration was 1,245.2 days in the magnesium-deficiency cohort and 1,350.0 days in the reference cohort; the corresponding median follow-up durations were 1,296 days (interquartile range, 797 days) and 1,449 days (interquartile range, 787 days), respectively. The magnesium deficiency cohort was associated with a higher incidence of overall dementia than the reference cohort (2.12% vs. 1.77%, HR 1.39, 95% CI, 1.28–1.51; *P* < 0.001) (Table 2). The corresponding absolute risk difference was 0.35 percentage points, yielding an approximate number needed to harm of 286 over 5 years based on observed event proportions. The E-value for the point estimate was 2.13 and the E-value for the lower bound of the confidence interval was 1.88. Among the secondary outcomes, low magnesium status was associated with a higher risk of Alzheimer’s disease (HR 1.34; *P* = 0.005), vascular dementia (HR 1.50; *P* = 0.001), other dementia types (HR 1.38; *P* < 0.001), and mild cognitive impairment (HR 1.27; *P* < 0.001) (Table 2). Because these secondary endpoint analyses were not adjusted for multiple comparisons, the nominal *P*-values should be interpreted as exploratory and hypothesis-generating. Kaplan–Meier analysis showed that patients with low magnesium levels had significantly lower dementia-free survival compared with matched controls over the 5-year observation period (Figure 2).

**TABLE 2:** Association between low magnesium status and 5-year risk of incident dementia after propensity score matching.

Outcome	Low Mg group (*n* = 58,991)	Control group (*n* = 58,991)	HR (95% CI)	*p*-value
	Events (%)	Events (%)		
Primary outcome				
Overall dementia	1,250 (2.12%)	1,044 (1.77%)	1.39 (1.28–1.51)	< 0.001
Secondary outcomes				
Vascular dementia	168 (0.29%)	129 (0.22%)	1.50 (1.19–1.89)	0.001
Other type of dementia	1,166 (1.98%)	979 (1.66%)	1.38 (1.27–1.50)	< 0.001
Alzheimer’s disease	195 (0.33%)	170 (0.29%)	1.34 (1.09–1.65)	0.005
Mild cognitive impairment	488 (0.83%)	449 (0.76%)	1.27 (1.12–1.44)	< 0.001

Mg, magnesium; HR, hazard ratio; CI, confidence interval.

### 3.3 Validation of study design

The positive control analysis demonstrated that patients with low magnesium status had a higher rate of subsequent hypocalcemia (HR 1.25; *P* < 0.001), consistent with the established physiological links (Table 3). The negative control outcome, acute appendicitis, showed no significant between-group differences (HR 0.86; *P* = 0.105). The healthcare visit rate was slightly higher in the magnesium deficiency cohort than in the reference cohort (HR 1.01; *P* = 0.03). The repeat COVID-19 infection rate was slightly lower in the magnesium deficiency cohort (HR 0.98; *P* = 0.016) (Table 3).

**TABLE 3:** Control outcomes, healthcare utilization, and repeat COVID-19 infection in the low magnesium analysis.

Outcome	Low Mg group (*n* = 58,991)	Control group (*n* = 58,991)	HR (95% CI)	*p*-value
	Events (%)	Events (%)		
Positive control outcome				
Serum calcium < 8.5 mg/dL	30,516 (51.73%)	28,188 (47.78%)	1.25 (1.23–1.27)	< 0.001
Negative control outcome				
Appendicitis	215 (0.36%)	274 (0.46%)	0.86 (0.72–1.03)	0.105
Healthcare utilization validation				
Healthcare visit^‡^	55,052 (93.32%)	56,327 (95.48%)	1.01 (1.00–1.03)	0.03
COVID-19 reinfection during follow-up				
Repeat COVID-19 infection	32,957 (55.87%)	35,033 (59.39%)	0.98 (0.97–1.00)	0.016

CI, confidence interval; COVID-19, coronavirus disease 2019; HR, hazard ratio; Mg, magnesium. ^‡^Healthcare visit was analyzed as a time-to-event outcome; therefore, the HR reflects the relative instantaneous rate of the first recorded follow-up visit and may differ from crude cumulative event proportions because of differences in visit timing, follow-up duration, and censoring.

### 3.4 Sensitivity analyses and subgroup analyses

The association between low magnesium status and incident dementia remained consistent across all three sensitivity analyses (Table 4). The point estimate was highest among patients admitted to the ICU following COVID-19 (HR 1.49; *P* < 0.001), followed by the analysis restricted to patients older than 65 years (HR 1.43; *P* < 0.001) and the analysis excluding patients who died during follow-up (HR 1.30; *P* < 0.001).

**TABLE 4:** Sensitivity analyses of the association between low magnesium status and 5-year risk of incident dementia.

Outcomes	Model I (*n* = 15,580 for each group)		Model II (*n* = 55,341for each group)		Model III (*n* = 39,357 for each group)	
	HR (95% CI)	*P*-value	HR (95% CI)	*P*-value	HR (95% CI)	*P*-value
Overall dementia	1.49 (1.27–1.74)	< 0.001	1.30 (1.19–1.43)	< 0.001	1.43 (1.31–1.56)	< 0.001
Vascular dementia	1.32 (0.86–2.02)	0.200	1.30 (1.01–1.66)	0.039	1.40 (1.10–1.78)	0.005
Other type of dementia	1.51 (1.28–1.78)	< 0.001	1.32 (1.20–1.45)	< 0.001	1.45 (1.32–1.59)	< 0.001
Alzheimer’s disease	1.62 (1.05–2.50)	0.029	1.20 (0.97–1.49)	0.094	1.29 (1.04–1.60)	0.023
Mild cognitive impairment	1.09 (0.85–1.40)	0.483	1.10 (0.97–1.25)	0.150	1.20 (1.04–1.38)	0.014

Model I was restricted to patients admitted to the intensive care unit during the COVID-19 episode. Model II excluded patients who died during follow-up. Model III was restricted to patients aged > 65 years. Hazard ratios were estimated using cause-specific Cox proportional hazards models. CI, confidence interval; COVID-19, coronavirus disease 2019; HR, hazard ratio; ICU, intensive care unit.

In subgroup analyses stratified by sex, low magnesium status was associated with a higher risk of overall dementia in both males (HR 1.51; *P* < 0.001) and females (HR 1.34; *P* < 0.001), with no statistically significant interaction between sex and magnesium status (P for interaction = 0.180; Table 5).

**TABLE 5:** Sex-stratified subgroup analyses of the association between low magnesium status and dementia risk.

Outcomes	Male (*n* = 27,484)		Female (*n* = 31,496)		P for interaction
	HR (95% CI)	*P*-value	HR (95% CI)	*P*-value	
Overall dementia	1.51 (1.32–1.73)	< 0.001	1.34 (1.21–1.49)	< 0.001	0.180
Vascular dementia	1.68 (1.18–2.40)	0.004	1.31 (0.98–1.76)	0.073	0.317
Other type of dementia	1.51 (1.31–1.73)	< 0.001	1.38 (1.24–1.53)	< 0.001	0.318
Alzheimer’s disease	1.14 (0.80–1.64)	0.468	1.16 (0.91–1.47)	0.227	0.938
Mild cognitive impairment	1.23 (1.01–1.51)	0.041	1.24 (1.05–1.46)	0.011	0.952

HR, hazard ratio; CI, confidence interval.

### 3.5 Adjusted hazard ratios from multivariable cox regression

Sequential multivariable Cox regression models confirmed the robustness of this association (Table 6). In the minimally adjusted model, controlling for age, sex, and race/ethnicity, the adjusted HR was 1.56 (*P* < 0.001). The progressive incorporation of cardiometabolic risk factors, systemic comorbidities, neurological and psychiatric conditions, and medication exposures attenuated the estimate modestly, with the fully adjusted model yielding an adjusted HR of 1.43 (*P* < 0.001).

**TABLE 6:** Sequential multivariable Cox regression models evaluating the association between low magnesium status and incident dementia.

Model	Adjusted HR (95% CI)	*p*-value	Covariates included in the model
Model 1	1.56 (1.46–1.65)	< 0.001	Age at index, sex, race/ethnicity
Model 2	1.46 (1.37–1.55)	< 0.001	Model 1 + cardiometabolic factors (hypertension, diabetes mellitus, overweight/obesity, nicotine dependence)
Model 3	1.44 (1.35–1.53)	< 0.001	Model 2 + systemic comorbidities (chronic kidney disease, liver disease, heart failure, chronic obstructive pulmonary disease, malnutrition, anemia)
Model 4	1.43 (1.34–1.53)	< 0.001	Model 3 + neurologic/psychiatric factors (cerebrovascular disease, intracranial injury, mood disorders, anxiety-related disorders)
Model 5	1.43 (1.35–1.54)	< 0.001	Model 4 + drug (antidepressants, CNS medications, diuretics, anticonvulsants, proton pump inhibitors)

CI, confidence interval; CNS, central nervous system; HR, hazard ratio; Mg, magnesium.

## 4 Discussion

In this large propensity score–matched cohort study of hospitalized COVID-19 survivors, low serum magnesium levels were associated with a higher risk of incident dementia over a 5-year follow-up period after a 1-year landmark. The association between magnesium deficiency and overall dementia remained consistent across sensitivity analyses, sex-stratified subgroups, and sequential multivariate models. These findings extend the limited existing evidence on potentially modifiable factors associated with post-COVID neurocognitive outcomes among COVID-19 survivors.

Our findings are broadly consistent with those of prior general-population studies linking low serum magnesium levels to dementia or cognitive impairment (18–22). However, these studies did not specifically consider antecedent infectious exposures or post-infectious neurodegenerative vulnerability. The only prior study directly examining magnesium status in relation to post-COVID cognitive outcomes was conducted by Guzmán-Esquivel et al. (29), who reported that hypomagnesemia predicted cognitive impairment among 111 older adults followed for 6 months. Our study extends this preliminary evidence by using a substantially larger cohort, longer follow-up, and repeated measurement exposure definition. A previous study also reported U-shaped associations between serum magnesium and dementia risk (21), suggesting that elevated magnesium levels may also be clinically relevant. We did not evaluate hypermagnesemia in the present analysis because, in routine clinical practice, elevated serum magnesium levels often reflect advanced kidney dysfunction or iatrogenic exposure rather than a stable nutritional state (33–35). This makes it difficult to isolate the independent contribution of high magnesium levels to the long-term risk of neurodegenerative diseases.

From a mechanistic perspective, magnesium is involved in synaptic plasticity, N-methyl-D-aspartate receptor regulation, and modulation of neuroinflammatory pathways (14–17). These functions may be particularly relevant when magnesium deficiency is superimposed on COVID-19–related endothelial dysfunction and microvascular injury. Consistent with this possibility, vascular dementia showed the largest point estimate among dementia subtypes in our analysis (HR 1.50), a finding that aligns with the role of magnesium in endothelial homeostasis and the recognized cerebrovascular complications of COVID-19 (11, 13, 27). However, all dementia subtype findings, including vascular dementia and Alzheimer’s disease, should be interpreted as exploratory because the event numbers were smaller than for overall dementia and no adjustment for multiplicity was applied. Similarly, the secondary endpoints, sensitivity analyses, and subgroup analyses were intended to assess consistency rather than provide confirmatory evidence, and their nominal *P*-values should not be overinterpreted in the absence of multiplicity adjustment.

Although the relative association was statistically robust, the absolute risk difference was small, corresponding to an approximate number needed to harm of 286 over 5 years; therefore, the clinical magnitude should be interpreted cautiously. In addition, the magnesium-deficiency cohort had a shorter mean follow-up duration than the reference cohort by approximately 105 days, which may reflect informative censoring related to mortality, loss to follow-up, or greater clinical instability. This shorter follow-up could have reduced the opportunity for dementia ascertainment in the magnesium-deficiency group and may therefore have led to underestimation, rather than overestimation, of dementia incidence in this cohort. Accordingly, both the small absolute risk difference and the unequal follow-up duration should be considered when interpreting the clinical significance of the observed association.

The prespecified control analyses supported the internal validity of the study design. Hypocalcemia, the positive control outcome, occurred more frequently in the magnesium-deficiency group, consistent with established physiological interactions between magnesium and calcium homeostasis. In contrast, acute appendicitis, the negative control outcome, showed no significant between-group difference, as expected. Healthcare visit rates were statistically higher in the magnesium-deficiency group (HR 1.01; *P* = 0.03); however, the magnitude of this difference was minimal and likely of limited clinical relevance. Nevertheless, differential surveillance cannot be fully excluded as a potential contributor to the observed association. Repeat COVID-19 infection was slightly less frequent in the magnesium-deficiency cohort (HR 0.98), suggesting that a greater reinfection burden was unlikely to account for the higher incidence of dementia.

The sensitivity and subgroup analyses provided additional context for interpreting the primary finding. The strongest association was observed among patients admitted to the ICU after COVID-19 (HR 1.49), which may reflect more severe neurological injury related to critical illness, including hypoxia, systemic inflammation, and sedative exposure. However, this interpretation warrants caution because ICU-admitted patients also have greater comorbidity burden and more complex pharmacologic exposures, limiting causal attribution. When patients who died during follow-up were excluded, the HR was attenuated to 1.30. This reduction may reflect conditioning on survival, because excluding deaths can disproportionately remove the most vulnerable individuals from the magnesium deficiency group and create a selected, relatively healthier analytic cohort. Importantly, the association remained statistically significant in both the primary and survival-restricted analyses.

The association was also consistent among patients older than 65 years and in both men and women, with no evidence of significant sex-based effect modification, supporting the robustness of the findings across clinically relevant subgroups. In sequential multivariable Cox models, the adjusted HR decreased only modestly from 1.56 to 1.43 after progressive adjustment, suggesting that measured confounders did not fully explain the association. The E-value for the primary endpoint was 2.13, with a lower confidence-limit E-value of 1.88, indicating that an unmeasured confounder would need to be associated with both magnesium deficiency and dementia by approximately twofold to fully account for the observed association. However, the E-value should be interpreted as a sensitivity metric rather than evidence that unmeasured confounding has been eliminated. The observed association may still reflect unmeasured frailty, nutritional status, medication burden, healthcare access, or social determinants of health rather than a direct effect of magnesium deficiency itself.

The study period spanned 2020–2023, during which the predominant SARS-CoV-2 variants and clinical management of COVID-19 changed substantially. Differences in viral neurotropism, acute disease severity, vaccination status, antiviral use, and hospitalization thresholds across pandemic phases may have contributed to heterogeneity in post-COVID neurocognitive risk (36, 37). Because variant-specific information was not reliably available in TriNetX, we could not determine whether the magnesium–dementia association differed across temporal or variant periods.

Several limitations should be acknowledged. First, as an observational study, the present analysis cannot establish causality; residual confounding from unmeasured variables such as apolipoprotein E genotype, dietary intake, and socioeconomic status may have biased the observed association in either direction. Clinically, low magnesium status may also be a marker of frailty, poor nutritional reserve, multimorbidity, polypharmacy, or unequal access to care, rather than a direct causal factor in dementia development. Therefore, the adjusted association should not be interpreted as evidence that magnesium correction would necessarily reduce dementia risk. Second, although COVID-19 could theoretically have contributed to persistent magnesium depletion, the 6-month interval and repeated-measurement design make it unlikely that the observed association is solely attributable to post-infectious metabolic disturbance. Third, the “repeatedly low” definition based on two measurements cannot capture the full magnesium trajectory, potentially misclassifying patients whose levels normalized between measurements and attenuating the true association. Fourth, serum magnesium was measured in routine clinical settings; patients selected for testing may have been systematically different from those who were not, potentially inflating the observed association through selection bias. Because study eligibility required two magnesium measurements, the analytic cohort represents a selected subgroup of hospitalized COVID-19 survivors with sufficient clinical contact to undergo repeated laboratory testing, which may limit generalizability to all COVID-19 survivors. In addition, although the absolute difference in follow-up healthcare utilization was small, the marginally higher visit rate in the low-magnesium group raises the possibility of differential dementia ascertainment. Fifth, ICD-10-CM–based dementia ascertainment likely underestimates true incidence, which would bias results toward the null. Sixth, the absence of data on magnesium supplementation dosage and standardized cognitive assessments precluded evaluation of dose–response relationships and detection of subclinical cognitive decline. Although magnesium supplementation was included as a baseline covariate and was well balanced after propensity score matching, TriNetX medication records may not reliably capture the indication, dose, duration, adherence, route of administration, or biochemical response to supplementation. Therefore, we could not validly evaluate magnesium supplementation as an effect modifier or determine whether magnesium correction attenuated the association between low magnesium status and incident dementia. Finally, the 1-year landmark design, while necessary to minimize protopathic bias from pre-existing but delayed-coded dementia, introduces survivorship bias by restricting the analytic cohort to patients who remained alive and dementia-free throughout the landmark period. In addition, death is an important competing event in this older post-hospitalization population. Although the cause-specific Cox model was appropriate for evaluating etiologic associations, it does not directly estimate the cumulative incidence of dementia in the presence of competing mortality. Given that approximately 6% of patients died during follow-up, treating death as censoring may have modestly overestimated cumulative dementia incidence if the results are interpreted as absolute risks. Therefore, the findings should be interpreted as conditional associations among patients who survived and remained under observation, rather than as population-level predictions of dementia risk.

## 5 Conclusion

In this large observational study, low serum magnesium levels were associated with a higher risk of incident dementia among hospitalized COVID-19 survivors over 5 years of follow-up. This association was consistent across sensitivity analyses, sex-stratified subgroups, and sequential multivariable models. Given the inherent limitations of observational design, these findings should be regarded as hypothesis-generating. Prospective studies with standardized cognitive assessments are warranted to validate this association and to clarify whether low magnesium status is a causal risk factor, a marker of frailty or poor nutrition, or both.

## Data Availability

The datasets presented in this article are not readily available because the patient-level source data are maintained within the TriNetX Global Collaborative Network and were not directly accessible to or exportable by the authors. Access to TriNetX data requires a direct agreement with TriNetX. De-identified aggregate outputs, cohort definitions, and post-export statistical code may be made available from the corresponding author upon reasonable request, where permitted by applicable data-use terms and institutional policies. Requests to access the datasets should be directed to https://live.trinetx.com.
