# Supplementary material for: Magnesium deficiency and long-term incident dementia among COVID-19 survivors: a propensity score-matched cohort study
Source: Front Nutr. 2026 Jul 1;13:1871623. doi: 10.3389/fnut.2026.1871623 (PMC13368526; doi:10.3389/fnut.2026.1871623)
Supplement: Supplementary file 1 [file Table_1.DOCX]

**Supplemental Table 1. Codes used for propensity score matching**

| Variable | Code / Definition |
| --- | --- |
| Age at index | Age ≥55 years |
| Sex | Female |
| Race/ethnicity | White: 2106-3; Black or African American: 2054-5; Asian: 2028-9 |
| Body mass index | BMI ≥30 kg/m²; TriNetX: 9083 |
| Essential hypertension | ICD-10-CM: I10 |
| Diabetes mellitus | ICD-10-CM: E08–E13 |
| Overweight and obesity | ICD-10-CM: E66 |
| Nicotine dependence | ICD-10-CM: F17 |
| Ischemic heart diseases | ICD-10-CM: I20–I25 |
| Chronic kidney disease | ICD-10-CM: N18 |
| Alcohol-related disorders | ICD-10-CM: F10 |
| Cerebrovascular diseases | ICD-10-CM: I60–I69 |
| Chronic obstructive pulmonary disease | ICD-10-CM: J44 |
| Malnutrition | ICD-10-CM: E40–E46 |
| Factors influencing health status and contact with health services | ICD-10-CM: Z00–Z99 |
| Sleep disorders | ICD-10-CM: G47 |
| Heart failure | ICD-10-CM: I50 |
| Atrial fibrillation and flutter | ICD-10-CM: I48 |
| Systemic connective tissue disorders | ICD-10-CM: M30–M36 |
| Mood disorders | ICD-10-CM: F30–F39 |
| Anxiety and related nonpsychotic mental disorders | ICD-10-CM: F40–F48 |
| COVID-19 | ICD-10-CM: U07.1 |
| Diseases of liver | ICD-10-CM: K70–K77 |
| Disorders of thyroid gland | ICD-10-CM: E00–E07 |
| Vitamin D deficiency | ICD-10-CM: E55 |
| Neoplasms | ICD-10-CM: C00–D49 |
| Intracranial injury | ICD-10-CM: S06 |
| COVID-19 vaccination | CPT: 0001A, 0002A, 0003A |
| Benzodiazepines / sedatives / hypnotics | TriNetX: CN302 |
| Insulins and analogues | ATC: A10A |
| Blood glucose-lowering drugs, excluding insulins | ATC: A10B |
| Central nervous system medications | TriNetX: CN000 |
| Diuretics | TriNetX: CV700 |
| Anticholinergics | ATC: S01FA |
| Anticonvulsants | TriNetX: CN400 |
| Magnesium supplementation | TriNetX: TN460 |
| Proton pump inhibitors | ATC: A02BC |
| Albumin | TriNetX: 9045; ≥3.5 g/dL |
| eGFR | LOINC: 98979-8; ≥60 mL/min/1.73 m² |
| HbA1c | TriNetX: 9037; ≥9% |
| C-reactive protein | TriNetX: 9063; ≥10 mg/L |
| Hemoglobin | TriNetX: 9014; ≥12 g/dL |
| Vitamin B12 | TriNetX: 9065; 300–900 pg/mL |
